# Incidence of adhesive capsulitis of the shoulder during the beginning of the COVID-19 pandemic

**DOI:** 10.31744/einstein_journal/2022AE0163

**Published:** 2022-11-18

**Authors:** Adham do Amaral e Castro, Renato Masson de Almeida Prado, Eduardo da Frota Carrera, Patrícia Yokoo, Durval do Carmo Barros Santos, Laercio Alberto Rosemberg, Atul Kumar Taneja

**Affiliations:** 1 Hospital Israelita Albert Einstein São Paulo SP Brazil Hospital Israelita Albert Einstein, São Paulo, SP, Brazil.

**Keywords:** Magnetic resonance imaging, COVID-19; Coronavirus infections, Betacoronavirus, SARS-CoV-2, Bursitis, Shoulder

## Abstract

**Objective:**

Adhesive capsulitis is an inflammatory disease of the joint capsule, clinically manifested as pain, stiffness, and dysfunction of the shoulder. We subjectively observed an increased incidence of adhesive capsulitis, and raised the hypothesis that adhesive capsulitis was more frequent in magnetic resonance imaging examinations performed during the COVID-19 pandemic as compared with examinations prior to this period.

**Methods:**

Data from medical records and magnetic resonance imaging of the shoulder presenting typical imaging findings of adhesive capsulitis, performed in our organization from March to June 2020, were evaluated and compared with data and imaging from the same period of the previous year. To this end, an organizational business intelligence tool called “search reports” was used, searching for the term “adhesive capsulitis” in the radiological report, results were tabulated, and corresponding magnetic resonance imaging exams were analyzed.

**Results:**

Our search found a total of 240 and 1,373 cases of adhesive capsulitis in the 2020 and 2019 periods, respectively. The mean age of patients was 53.9 years in the 2020 group and 49.9 years in 2019 (p<0.001). Magnetic resonance imaging findings were positive for adhesive capsulitis in 40 out of 240 shoulders (16.7%) in the 2020 group *versus* 127 out of 1,373 shoulders (9.2%) in the 2019 group. This difference was statistically significant (p=0.001).

**Conclusion:**

Our study findings suggest a relative increase in the proportion of magnetic resonance imaging findings suggestive of adhesive capsulitis cases during COVID-19 pandemics based on data from our organization.

## INTRODUCTION

Adhesive capsulitis, also called “frozen shoulder,” is an inflammatory disease of the joint capsule, clinically manifested as pain, stiffness, and dysfunction of the shoulder. It typically affects middle-aged women and is associated with systemic diseases, such as obesity, *diabetes mellitus*, and hypothyroidism.^(1,2)^

Diagnosis of adhesive capsulitis is clinical, and manifestations include shoulder pain and reduced range of motion, in a setting without no other explanation for the symptoms. Imaging diagnosis of adhesive capsulitis is made mainly by magnetic resonance imaging (MRI), with good accuracy. Findings suggesting adhesive capsulitis on MRI include coracohumeral ligament thickening, fat obliteration of the rotator interval (RI), RI enhancement, axillary joint capsule thickening and enhancement, and inferior glenohumeral ligament thickening and hyperintensity.^(3,4)^

Coronavirus disease 2019 (COVID-19) was officially declared a pandemic by the World Health Organization (WHO) in 2020. This disease spread worldwide and is caused by a novel virus known as severe acute respiratory syndrome coronavirus 2 (SARS-CoV-2). By November 2021, over 250 million cases of COVID-19 had occurred, with over 5 million deaths reported.^(5)^The orthopedical practice has also been affected by the pandemic, mainly due to social restriction and lockdown policies to control disease transmission. A marked reduction in traumatological diseases and a decline in outpatient visits have been reported in many organizations.^(6)^

Although COVID-19 is primarily a respiratory disease, multisystem involvement has been reported. Musculoskeletal manifestations of COVID-19 are rare and include muscle, nerve, joint, soft tissue, and bone involvement.^(7)^ Recently, Ascani et al. reported 12 cases of adhesive capsulitis in patients after COVID-19 infection.^(8)^

In our organization, we subjectively noted an increase in prevalence of adhesive capsulitis, both in MRI examinations and in outpatient consultations.

## OBJECTIVE

To assess the hypothesis raised that adhesive capsulitis was more frequent during the COVID-19 pandemic as compared with a prior period.

## METHODS

### Materials

This study was approved by the Institutional Review Board of *Hospital Israelita Albert Einstein* (CAAE: 38424820.6.0000.0071; # 4.572.036) and informed consent was waived due to its retrospective nature.

Data from medical records and MRI of the shoulder presenting typical imaging findings of adhesive capsulitis, performed in our organization from March to June 2020 (corresponding to the first 4 months of the pandemic in Brazil), were evaluated and compared with data and imaging from the same period of the previous year. To this end, an organizational business intelligence tool called “search reports” was used, searching for the term “adhesive capsulitis” in the radiological report.

Each subject had their anthropometric, clinical, and imaging findings gathered and tabulated, as follow:

### Anthropometric data

Sex, age, weight, height, body mass index.

### Clinical data

Presence and duration of pain symptoms, movement limitation and any other symptoms mentioned. Presence of comorbidities reported by the patient.

### Adhesive capsulitis related imaging data

Cases were included in the study if they presented the diagnosis of adhesive capsulitis in the radiological report. Such diagnosis is suggested in our reports when at least one of the following findings are present: glenohumeral capsular thickening, pericapsular edema, edema of the adipose planes in the rotator interval, thickening of the coracohumeral ligament. These exam findings were reported in absolute and relative terms (proportion of shoulder MRI diagnosed with capsulitis in relation to the total shoulder MRI performed for the period evaluated).

### Statistical analysis

Patients’ age was described using summary measures (mean, standard deviation, median, minimum, and maximum). Absolute and relative frequencies, as well as the association, were evaluated by *χ*^2^ test and the Student´s *t* test. IBM-SPSS for Windows version 22.0 software was used to perform the analyses, and Microsoft Excel 2010 software to tabulate data. The tests were performed with a significance level of 5%.

## RESULTS

The totals of 1,373 and 240 shoulder MRI examinations were performed, respectively, from March to June 2019, and from March to June 2020, and this was the database for the present study. No patients were excluded from this study.

### Demographics

Patients in the 2020 group had a mean age of 53.9 years (range 18-88 years), and in the 2019 group, 49.9 years (range 6-93 years). This difference was statistically significant (p<0.001). The age distribution is summarized in table 1.

**Table 1:** Age distribution

Year	Mean	SD	Median	Minimum	Maximum	Total	p value
2019	49.9	14.3	50	6	93	1,373	<0.001
2020	53.9	14.4	54.5	18	88	240	
Total	50.5	14.4	50	6	93	1,613	

SD: standard deviation.

In the 2020 group, 111 (46.2%) patients were female and 129 (53.8%) were male. In the 2019 group, there were 619 (45.1%) female patients and 754 (54.9%) males. This difference in sex distribution was not statistically significant (p=0.732). Sex distribution is summarized in table 2.

**Table 2:** Sex distribution

	2019 n (%)	2020 n (%)	Total n (%)	p value
Female	619 (45.1)	111 (46.2)	730 (45.3)	0.732
Male	754 (54.9)	129 (53.8)	883 (54.7)	
Total	1,373 (100)	240 (100)	1,613 (100)	

### Adhesive capsulitis

**Figure 1:**
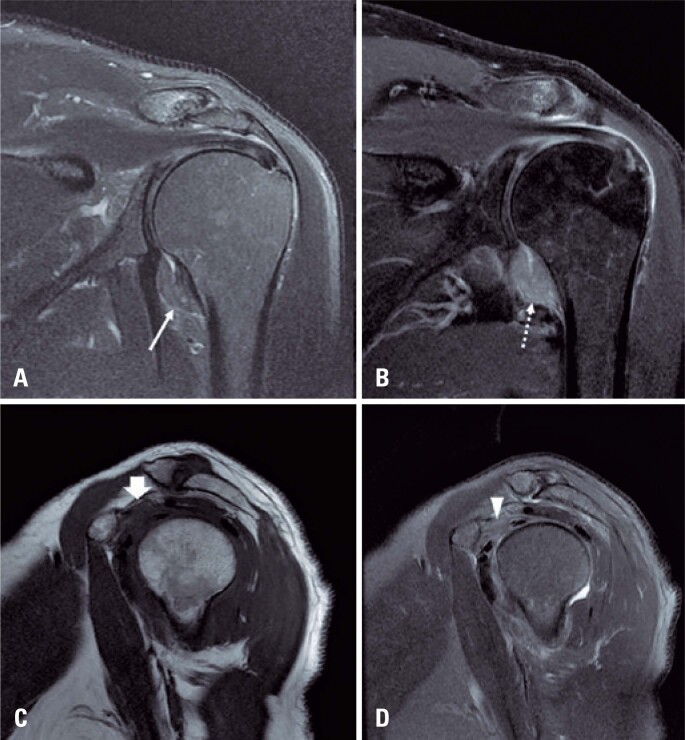
Typical case of adhesive capsulitis in a 64-year-old male patient with shoulder pain for one month, without trauma. Coronal T2 fat suppression-weighted-image (A) shows glenohumeral capsular thickening and pericapsular edema affecting the axillary recess (arrow). Coronal T1-weighted-image post gadolinium with fat suppression (B) shows glenohumeral capsular thickening and capsular and pericapsular enhancement affecting the axillary recess (dashed arrow). Sagittal T1-weighted-image (C) and T2-weighted-image with fat suppression (D) shows obliteration of the pericapsular fat tissue along the rotator interval region (short arrow and arrowhead)

In the 2020 group, 40 out of 240 shoulders (16.7%) had MRI findings consistent with adhesive capsulitis (Figure 1). In the 2019 group, 127 out of 1,373 shoulders (9.2%) had positive MRI findings of adhesive capsulitis. This difference was statistically significant (p=0.001). The difference between the two groups is summarized in table 3.

**Table 3:** Prevalence of adhesive capsulitis according to magnetic resonance imaging criteria

	2019 n (%)	2020 n (%)	Total n (%)	p value
Negative	1,246 (90.8)	200 (83.3)	1,446 (89.6)	0.001
Positive	127 (9.2)	40 (16.7)	167 (10.4)	
Total	1,373 (100)	240 (100)	1,613 (100)	

## DISCUSSION

COVID-19 was declared a pandemic in March 2020, and some interventions have been employed to stop dissemination of the disease, including mask-wearing policies and social isolation. It is reported that these measures, as well as the disease itself, have changed the daily practice in orthopedic centers^(6)^ and led to change in number of cases, surgeries and diagnoses.

Adhesive capsulitis is an inflammatory condition of the shoulder, manifested clinically as pain and reduced range of motion.^(3,4)^ Although the pathogenesis of disease is uncertain, some risk factors have been identified, such as obesity, hypothyroidism, and diabetes.^(1,2)^ In a study by Ebrahimzadeh et al., anxiety and depression were associated with a higher risk of pain in patients with adhesive capsulitis.^(9)^ In a letter to the editor, Vitali et al.^(10)^ also observing an increased prevalence of adhesive capsulitis during the COVID-19 pandemic, suggested three mechanisms: lack of appropriate physical therapy, leading to painful rigidity of the shoulder; depression and/or anxiety due to COVID-19 pandemic; and relation to extrapulmonary manifestations of COVID-19.

**Figure 2:**
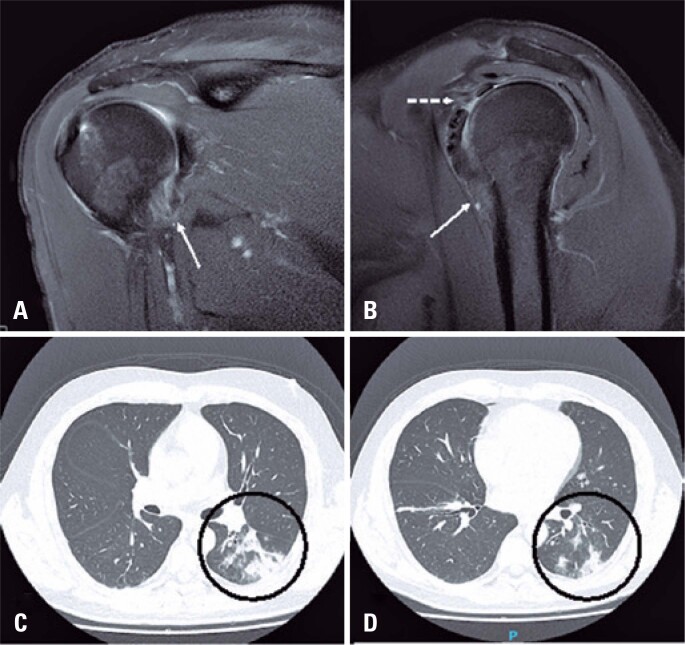
(A) A 48-year-old male patient submitted to magnetic resonance imaging study of the right shoulder with recent symptoms of pain and limited range of movement, almost simultaneously with diagnosis of COVID-19. Magnetic resonance imaging showed findings consistent with adhesive capsulitis, with capsular thickening (solid arrows in A and B) and pericapsular edema affecting the rotator interval (dashed arrow in B). During the same period, the patient was admitted to hospital due to shortness of breath and decreased oxygen saturation, and was diagnosed as COVID-19. A computed tomography scan of the chest revealed areas of ground glass opacities and alveolar opacification in the left lower lobe (circles in C and D)

COVID-19 is primarily a respiratory disease, and the most common cause for intensive-care-unit admission is hypoxemic respiratory failure.^(11)^ Extrapulmonary manifestations have been reported, including gastrointestinal symptoms, kidney and liver injury, myocardial dysfunction, endocarditis, acute coronary syndrome, neurologic complications, and dermatologic findings.^(7,11)^ Musculoskeletal manifestations of COVID-19 are rare and include muscle, nerve, joint, soft tissue, and bone involvement.^(7)^ These can be caused by direct infection by SARS-CoV-2, immune-mediated mechanisms, and iatrogenesis. For example, prone positioning for optimization of oxygenation is a risk factor for compressive injury of the brachial plexus.^(7,12)^ Therefore, it is plausible to think that social isolation measures can have adverse effects and contribute to increased incidence of adhesive capsulitis, as suggested by Vitali et al,^(10)^ or even an auto-immune effect of the viral infection itself (Figure 2). In the first 4 months of the pandemic less knowledge about the disease, restrictive measures in place, and scarce information about and availability of vaccines were factors may have had an important influence on the results found in the present study. We did not have full access to data on concomitant or prior infection of COVID-19 with shoulder adhesive capsulitis, and this is an interesting aspect to investigate in future studies.

Our study has some limitations. First, its report-based search retrospective nature. Second, although clinical assessment is the gold standard for diagnosis of adhesive capsulitis, we did not have this data available for all cases. This would require a different methodology, and we would not be able to enroll as many patients; thus, we relied on imaging findings consistent with adhesive capsulitis. Moreover, there are some biases. For example, occupational and sports-related issues could be less frequent during the pandemic due to social isolation measures, and there was a relative increase in adhesive capsulitis, without an actual increase in number of cases. Prospective studies during the pandemics would be helpful to clarify this issue.

## CONCLUSION

In conclusion, our results show that there was an increase in the proportion of magnetic resonance imaging findings suggestive of adhesive capsulitis during the pandemic.

## References

[1] Ramirez J (2019). Adhesive capsulitis: diagnosis and management. Am Fam Physician.

[2] Kingston K, Curry EJ, Galvin JW, Li X (2018). Shoulder adhesive capsulitis: epidemiology and predictors of surgery. J Shoulder Elbow Surg.

[3] Suh CH, Yun SJ, Jin W, Lee SH, Park SY, Park JS (2019). Systematic review and meta-analysis of magnetic resonance imaging features for diagnosis of adhesive capsulitis of the shoulder. Eur Radiol.

[4] Fields BK, Skalski MR, Patel DB, White EA, Tomasian A, Gross JS (2019). Adhesive capsulitis: review of imaging findings, pathophysiology, clinical presentation, and treatment options. Skeletal Radiol.

[5] Johns Hopkins University & Medicine. Coronavirus Resource Center (2021). COVID-19 Dashboard by the Center for Systems Science and Engineering (CSSE) at Johns Hopkins University (JHU).

[6] Maniscalco P, Poggiali E, Quattrini F, Ciatti C, Magnacavallo A, Caprioli S (2020). The deep impact of novel COVID-19 infection in an orthopedics and traumatology department: the experience of the Piacenza Hospital. Acta Biomed.

[7] Ramani SL, Samet J, Franz CK, Hsieh C, Nguyen CV, Horbinski C (2021). Musculoskeletal involvement of COVID-19: review of imaging. Skeletal Radiol.

[8] Ascani C, Passaretti D, Scacchi M, Bullitta G, De Cupis M, Pasqualetto M (2021). Can adhesive capsulitis of the shoulder be a consequence of COVID-19? Case series of 12 patients. J Shoulder Elb Surg.

[9] Ebrahimzadeh MH, Moradi A, Bidgoli HF, Zarei B (2019). The relationship between depression or anxiety symptoms and objective and subjective symptoms of patients with frozen shoulder. Int J Prev Med.

[10] Vitali M, Pironti P, Salvato D, Salini V (2021). Can COVID-19 pandemic influence frozen shoulder outcomes?. Rehabilitation (Madr).

[11] Wiersinga WJ, Rhodes A, Cheng AC, Peacock SJ, Prescott HC (2020). Pathophysiology, transmission, diagnosis, and treatment of coronavirus disease 2019 (COVID-19): a review. JAMA.

[12] Miller C, O’Sullivan J, Jeffrey J, Power D (2021). Brachial plexus neuropathies during the COVID-19 pandemic: a retrospective case series of 15 patients in critical care. Phys Ther.

